# Depression patient-derived cortical neurons reveal potential biomarkers for antidepressant response

**DOI:** 10.1192/j.eurpsy.2021.392

**Published:** 2021-08-13

**Authors:** Y. Avior, S. Ron, D. Kroitorou, E. Nitzan, B. Corneo, D. Laifenfeld, T. Cohen Solal

**Affiliations:** 1 R&d, Genetika+, Jerusalem, Israel; 2 Stem Cell Core Facility, Columbia University, New York City, United States of America

**Keywords:** Depression, personalized medicine, biomarkers, disease models

## Abstract

**Introduction:**

Major depressive disorder is highly prevalent worldwide and has been affecting an increasing number of people each year. Current first line antidepressants show merely 37% remission, and physicians are forced to use a trial-and-error approach when choosing a single antidepressant out of dozens of available medications.

**Objectives:**

We sought to identify a method of testing that would provide patient-specific information on whether a patient will respond to a medication using in vitro modeling.

**Methods:**

Patient-derived lymphoblastoid cell lines from the STAR*D study were used to rapidly generate cortical neurons and screen them for bupropion effects, for which the donor patients showed remission or non-remission.

**Results:**

We provide evidence for biomarkers specific for bupropion response, including synaptic connectivity and morphology changes as well as specific gene expression alterations.

**Conclusions:**

These biomarkers support the concept of personalized antidepressant treatment based on in vitro platforms and could be utilized as predictors to patient response in the clinic.

**Disclosure:**

This work was funded by Genetika+ Ltd, Jerusalem, Israel. YA, DK, EN, DL and TCS are employees of Genetika+ Ltd and received salary and/or stock options for the submitted work.

